# Recovery of Polyphenols from Rosehip Seed Waste Using Natural Deep Eutectic Solvents and Ultrasonic Waves Simultaneously

**DOI:** 10.3390/foods12193655

**Published:** 2023-10-03

**Authors:** Aleksandra Gavarić, Kristian Pastor, Nataša Nastić, Senka Vidović, Nemanja Živanović, Nataša Simin, Ana Rita C. Duarte, Jelena Vladić

**Affiliations:** 1 Faculty of Technology, University of Novi Sad, Bulevar cara Lazara 1, 21000 Novi Sad, Serbia; cvejina@uns.ac.rs (A.G.); kristian.pastor@uns.ac.rs (K.P.); natasa.nastic@uns.ac.rs (N.N.);; 2 Faculty of Sciences, University of Novi Sad, Trg Dositeja Obradovića 3, 21000 Novi Sad, Serbia; nemanja.zivanovic@dh.uns.ac.rs (N.Ž.); natasa.simin@dh.uns.ac.rs (N.S.); 3 LAQV, REQUIMTE, Chemistry Department, NOVA School of Science and Technology, 2829-516 Caparica, Portugal

**Keywords:** rosehip seed, natural deep eutectic solvent, polyphenols, ultrasound extraction, principal component analysis

## Abstract

Rosehips are processed and consumed in numerous forms, such as juice, wine, herbal tea, yogurt, preserved fruit, and canned products. The seeds share in fruit is 30–35% and they have recently been recognized as an important source of oil rich in unsaturated fatty acids. However, after defatting, seed waste may still contain some polar polyphenolic compounds, which have been scarcely investigated. The aim of this study was to examine the potential of the defatted seed waste as a source of polyphenols. For the defatting process, supercritical carbon dioxide extraction at 300 bar and 40 °C was applied. The capacity of eight different natural deep eutectic solvents (NADES) for the recovery of phenolics from defatted rosehip seed powder (dRSP) was examined. In the extracts obtained with ultrasound-assisted NADES extraction, twenty-one phenolic compounds were identified with LC-MS/MS, among which the most abundant were quinic acid (22.43 × 10^3^ µg/g dRSP) and catechin (571.93 µg/g dRSP). Ternary NADES formulations based on lactic acid proved to be superior. Potential correlations between identified chemical compounds, solvent polarity and viscosity, as well as the compound distributions across studied solvent combinations in PCA hyperspace, were also investigated. PCA demonstrated that more polar NADES mixtures showed improved extraction potential. The established environmentally friendly process represents an approach of transforming rosehip seed waste into value-added products with the potential to be applied in the food industry and to contribute to sustainable production.

## 1. Introduction

Rosehip (*Rosa canina* L.) is a widely geographically represented plant species belonging to the family Rosaceae and genus Rosa. Due to its important nutritional and health aspects, rosehip is used in numerous production processes. It is most used in the production of personal care and cosmetic products, followed by pharmaceutical and nutraceutical products, food and beverage, and as animal feed [1]. In 2021, the rosehip extracts market size exceeded USD 340 million and is expected to exhibit a 9% compound annual growth rate (CAGR) through 2022–2030; while in Europe, the rosehip extracts market share is projected to have a CARG more than 9% by 2030 [1].

The use of pericarp parts in the production of various beverages (juices and teas) and food products such as jams, jellies, marmalades, yogurts, and other products is much more widespread. Rosehip seed, which constitutes 30–35% of the fruit is, however, less studied and, depending on the industry, is often discharged as waste material in production. Since it is an inexpensive source of unsaturated fatty acid-rich oil, the rosehip fruit seed has a great potential for valorization, particularly in cosmetic products such as skin care and anti-aging formulations [2]. Many studies have been conducted to date on the fatty acid composition of oil fraction of rosehip seed using different extraction methods and solvents [3,4,5]. Therefore, the use of rosehip oil has increased significantly, and market insights demonstrate that the rosehip oil market value was worth USD 150 million in 2021 [1]. It was found that besides being a good source of oil and fatty acids, the seed also contains phenolic components. Medveckienė et al. [6] established a significant presence of phenols in rosehip seed, and identified five phenolic acids (gallic, chlorogenic, caffeic, *p*-coumaric, and ferulic acid).

Therefore, after using the pericarp, the seed can be used for the attainment of oil, and after that the defatted seed residue can be used as a source of phenolic components. This procedure would represent a more complete utilization of the raw material and provide high value phenolic components from an inexpensive renewable source. The possibility of recovering polyphenolic components from defatted seed residue has not been previously investigated in the literature.

A new class of green solvents, deep eutectic solvents (DES), has attracted scientific attention worldwide. These solvents are defined as a mixture of two or more components, a hydrogen bond acceptor (HBA) and a hydrogen bond donor (HBD), combination of which upon mixing in particular molar ratios creates a mixture with an exceptionally low melting temperature, lower than that of any of their individual components [7,8]. If DES are prepared with naturally occurring components such as sugars, alcohols, amino acids, and so on, they are assigned as natural deep eutectic solvents (NADES). The driving force to produce NADES is the interaction of hydrogen bonding between the HBAs and the HBDs. The list of advantages of using NADES is extensive, including environmental friendliness, biodegradability, and non-toxicity which makes them adequate for application in food, pharmaceutical, and cosmetic products. In addition, depending on their application and safety issues, the extracts obtained with NADES do not require purification after extraction. Also, preserving the extracts in the NADES can increase their shelf life and bioactivity; thus, promoting their stability [9,10].

The aim of this study was to investigate the possibility of using defatted industrial rosehip seed waste as a source of polyphenolic components with the use of green solvents. The capacity of different NADES formulations in combination with ultrasonic-assisted extraction to extract polyphenols from defatted rosehip seed powder (dRSP) was evaluated. The approach of valorization of defatted rosehip seed as a source of polyphenols had not been previously investigated. Also, the polyphenolic profile of rosehip seed was analyzed in detail for the first time using liquid chromatography–tandem mass spectrometry. Furthermore, this study aimed to examine possible correlations between solvent polarity and viscosity and the isolation of specific chemical compounds. Additionally, this study sought to explore the extraction potential of different solvent combinations within a multivariate data space. By analyzing these relationships, we aimed to gain deeper insights into the interactions between solvent properties and the efficiency of extracting particular compounds, leading to a more comprehensive understanding of the extraction process of rosehip seed waste.

## 2. Materials and Methods

### 2.1. Plant Material

The defatted rosehip seed powder (dRSP) with less than 2500 µm of mean particle size was used for analysis. The rosehip seeds were discharged as waste from the filter tea factory “Fructus” (Backa Palanka, Serbia).

The rosehip seeds were spread in a thin layer in aluminum trays and vacuum dried in a vacuum oven (Kemoservis-Fotomaterial, Ljubljana, Slovenia) at 40 °C at an absolute pressure of 40 mbar for 10 h. After drying, the material was ground and sieved using the Retsch AS 200 Control sieving device (Retsch, GmbH, Haan, Germany).

Defatting of RSP was carried out using a supercritical carbon dioxide extraction (HPEP, NOVA-Swiss, Effretikon, Switzerland) under 300 bar, 40 °C, for 4 h.

### 2.2. Chemicals

Betaine anhydrous (≥99% purity), lactic acid (≥85% purity), L-proline (≥99% purity), D-glucose monohydrate (≥97.5% purity), D-menthol (≥95% purity), thymol (≥99% purity), and carvacrol (≥98% purity) were all purchased from Sigma–Aldrich (St. Louis, MO, USA), while glycerol (99.5% purity) was purchased from Scharlab (Sentmenat, Spain).

### 2.3. Preparation of NADES

NADES mixtures were produced in a water bath at 60 °C, except for LA:Pro which was prepared at ≤40 °C to avoid degradation, and placed on a magnetic stirrer hot plate. Mixing lasted until a homogeneous, transparent liquid was formed. All prepared NADES were stable and transparent at room temperature. NADES used in this work, abbreviations, and molar ratios are shown in Table 1.

### 2.4. Determination of Polarity

The polarity of the prepared NADES was determined using solvatochromic method with Nile red, described previously in Fernandes et al. [11]. The absorbance of the samples was obtained using a UV-spectrophotometer (GENESYS 50, Thermo Scientific, Waltham, MA, USA) wavelength range of 400–800 nm. Measurements were conducted in triplicates. Polarity of BE:Gly was determined previously [12].

### 2.5. Determination of Viscosity

The viscosity of DES was carried out using an MCR102 Modular Compact Rheometer (Anton Parr, Graz, Austria) fitted with a parallel plate geometry with 50 mm of diameter (PP50, Anton Parr, Graz, Austria). Measurements of viscosity of the systems were performed in the temperature range of 60–20 °C (2 °C/min). Measurements were conducted in triplicates. Viscosity of BE:Gly was determined previously [12]. NADES used in this work, abbreviations, molar ratios, polarities and viscosities at 60 °C are shown in Table S1.

### 2.6. Ultrasound-Assisted Extraction (UAE)

After the defatting process, dRSP was mixed with NADES and exposed to ultrasound-assisted extraction (UAE). In all UAE experimental runs, the solid/liquid ratio was 1:20 (*w*/*w*) and at a temperature of 60 °C with different extraction times (30, 60, and 90 min). UAE was performed in a sonication water bath (Grant Instruments, Royston, UK) with an ultrasonic power of 100 W, and frequency of 50–60 Hz. After the extraction, a supernatant was separated from solid residue by filtering through cotton wool in a 5 mL syringe, and stored in a glass container in a freezer until further analysis.

### 2.7. HPLC-MS-MS Analysis

The content of quinic acid, catechin, and 43 selected phenolic compounds (14 phenolic acids, 24 flavonoids, 3 coumarins, and 2 lignans) was determined with liquid chromatography–tandem mass spectrometry (LC-MS/MS) according to the previously reported method [13]. Standards of the compounds were purchased from Sigma–Aldrich Chem (Steinheim, Germany), Fluka Chemie GmbH (Buchs, Switzerland), or from ChromaDex (Santa Ana, CA, USA). Samples and standards were analyzed using Agilent Technologies 1200 Series high-performance liquid chromatograph coupled with Agilent Technologies 6410A Triple Quad tandem mass spectrometer with electrospray ion source and controlled with Agilent Technologies MassHunter Workstation software—data acquisition (ver. B.06.00) (Agilent Technologies, Santa Clara, CA, United States). The sample (5 μL) was injected into the system, and compounds were separated on Zorbax Eclipse XDB-C18 (50 × 4.6 mm, 1.8 μm) rapid resolution column which was thermostated at 50 °C, with mobile phase flow of 1 mL/min. Composition and gradient of mobile phase was A = 0.05% (*v*/*v*) formic acid, B = MeOH; 0 min 30% B, 6 min 70% B, 9 min 100% B, 12 min 100% B, and a post time of 3 min (total time of analysis 15 min). Data were acquired in dynamic MRM mode, and peak areas were determined using Agilent MassHunter Workstation Software—qualitative analysis (ver. B.06.00). Calibration curves were plotted with OriginLabs Origin Pro (ver. 2019b) software and used for calculation of investigated compounds concentration in the extracts. Results are expressed as microgram per gram of dry weight (µg/g dw). Validation results are given in Table S2 in Supplementary Materials. All the compounds that were present in the extracts were quantified; for the compounds that were present in a concentration lower than the lowest in the calibration, it was identified that they were lower than the relevant concentration. Compounds below quantification limit were given as <*LoQ*, where *LoQ* is a method quantification limit calculated from instrument quantification limit and sample dilution.

### 2.8. Statistical Analysis

Principal component analysis (PCA) was employed to reduce data dimensionality and identify data groups and to enhance interpretation of the obtained results. Two-dimensional PCA bi-plots were built on obtained extracts using different solvent combinations as scores, and detected chemical compounds, solvent polarity and solvent viscosity as loadings. Chemical compound concentrations expressed as µg/g dRSP, polarity (*E*_NR_) as kcal·mol^−1^ and viscosity (*η*) as mPa·s were applied. Since increasing values of *E*_NR_ (kcal·mol^−1^) indicate non-polarity, a value reduced from the value of 100 was applied to evaluate polarity. Moreover, the potential correlations between identified chemical compounds and solvent mixture properties—polarity and viscosity—were investigated by calculating Spearman’s correlation indices. The open-source Paleontological Statistics software (PAST v4.10) was applied for this purpose (Natural History Museum, University of Oslo, Norway).

All analyses were carried out in triplicate and the results were expressed as means ± standard deviation. Mean values were considered significantly different at a *p* < 0.05 confidence level after the performance of the one-way ANOVA statistical analysis followed by Tukey’s HSD post hoc test.

## 3. Results and Discussion

### 3.1. HPLC-MS-MS Analysis of Phenolics in R. canina Defatted Seed Extracts

**Figure 1:**
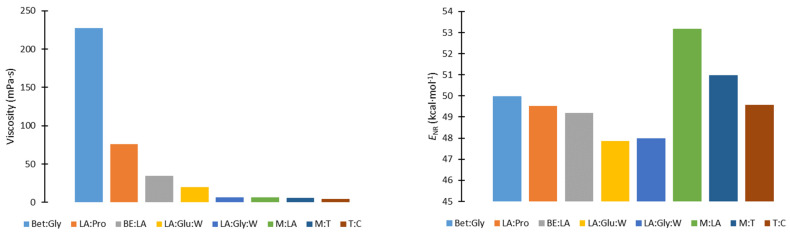
Viscosity and relative polarity (expressed as *E*_NR_ values) of NADES at temperature 60 °C (Table S1, Supplementary material).

After rosehip seed waste from the tea industry was provided, the defatting process was carried out using supercritical CO_2_ extraction at conditions of 300 bar and 40 °C, which were selected based on the study of Salgin et al. [14]. A yield of 5.42 ± 10% (*w*/*w*) was achieved. The obtained yield was in accordance with the literature; namely, Jakovljevic et al. [15] reported a yield between 3.26–7.75% with supercritical CO_2_ extraction. Bearing in mind that the chemical profile of rosehip seed was previously well-investigated [4,16], the composition of the oil was not analyzed in this study. Furthermore, the focus of this study was on the valorization of the remaining defatted rosehip and on determining the phenolic profile of extracts obtained using NADES. Since the properties of solvents such as polarity and viscosity are essential to modulate their solubilizing capabilities and their interactions with bioactives of interest, NADES with different characteristics (Figure 1; Table 1) based on betaine, lactic acid, and oxygenated monoterpenes were selected for extraction. NADES components and their molar ratios were selected based on the experience of the research group. In addition, lactic acid and betaine-based NADES have previously been confirmed to be efficient for the extraction of polyphenols [17,18]. Moreover, one of the criteria was the possibility of application of obtained extracts in food, pharmaceutical, and cosmetic products.

The phenolic content of seeds has been scarcely investigated in previous studies and only the total phenolic content, determined with spectrophotometric analysis, was mainly reported. According to Ilyasoğlu et al. [4], the rosehip seed extract obtained with methanol extraction (with shaking at 250 rpm, at room temperature, for 2 h) contained total phenolic content of 255 μg/g seed. Considering the total sum of identified components, which ranged from 20.52 to 22,799.03 µg/g dRSP (in our work), the phenolic content in the extracts obtained with ultrasonic-assisted NADES extraction was several times higher. The variability in terms of the chemical profile of plant material is common and may be explained due to different climatic, geographical characteristics, and methods of obtaining extracts. According to our knowledge, Medveckienė et al. [6] were the only ones to identify phenolic acids in seed extracts. Namely, they identified the presence of gallic, chlorogenic, caffeic, *p*-coumaric, and ferulic acid and found the total phenolic acid content to be 1.75 ± 0.05 mg/g of dry weight. 

The polyphenolic profiles of dRSP extracts obtained with ultrasonic-assisted NADES extraction at 60 °C at different extraction times (30, 60, and 90 min) were determined with liquid chromatography–tandem mass spectrometry (LC-MS/MS) (Table 2 and Table 3). The LC-MS/MS analysis of 44 compounds resulted in the identification of 21 compounds, among which were quinic acid, phenolic acids, flavonoids, and glycosides. Among the 21 identified, 19 were present in a concentration above the limit of quantification (LoQ).

NADES composition also determines the physicochemical characteristics of the system, which depends on the interaction with the target components and the efficiency of their extraction. Comparing the total sum of identified components, lactic acid-based solvents, which were the most polar solvents (with the lowest ENR value) and had the lowest viscosity, proved to be the most adequate for the recovery of phenolic components and quinic acid, while betaine-based (ENR—20.52-532.81) and terpene-based (ENR—224.76–451.63) solvents were less effective. Terpene-based solvents had the lowest viscosity and therefore the possibility of easy penetration into the material. However, their reduced polarity limited the efficiency of polyphenol extraction due to a lower affinity towards target components. On the other hand, betaine-based systems were more polar than the terpene-based but were more viscous, which made it difficult for them to diffuse into the material and interact with the components; thus, the least effective system was the most viscous system (BE:Gly).

The most abundant component in the extracts was quinic acid, and depending on the extraction conditions and solvent, its content ranged from 0.44 to 22,428.68 µg/g dRSP (BE:Gly for 30 min and LA:Glu:W for 90 min, respectively). According to the results of in vivo and in vitro studies, quinic acid represents a potentially important natural resource in drug development due to the numerous properties it possesses, such as antimicrobial, analgesic, antioxidant, antidiabetic, and anticancer properties [19]. Quinic acid was previously identified as the most abundant compound in water and methanolic extracts of *R. canina* and *R. arvensis* rosehips with seeds and traditionally prepared purée and jam extracts (0.27 ± 0.01 × 10^3^–4.52 ± 0.02 × 10^3^ μg/g dw) [20]. Also, *R. canina* fruit extract obtained with methanol extraction contained a significant concentration of quinic acid (1102.59 μg/g dw) [21].

**Figure 2:**
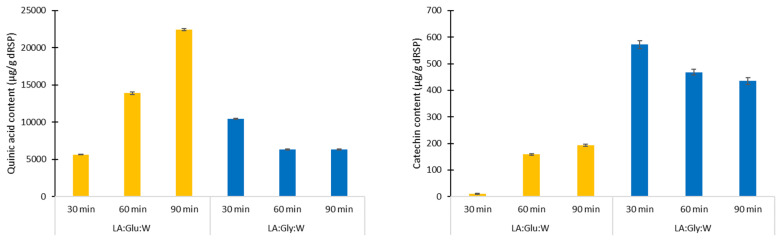
Content of quinine acid (left) and catechin (right) in LA:Glu:W (5:1:3) and La:Gly:W (3:1:3).

Lactic acid-based NADES were more adequate in the extraction of quinic acid (692.98–22428.68 µg/g dRSP) compared to betaine-based (0.44–267.44 µg/g dRSP) and terpene-based NADES (160.76–303.15 µg/g dRSP) (Table 2). By using a system where sugar alcohol is used instead of sugar, the efficiency of quinic acid extraction was reduced more than two times (Figure 2). When glucose was constituent of the ternary system, with increasing of the extraction time from 30 to 90 min, the extraction yield of quinic acid raised almost four-fold. When glycerol was a constituent of the ternary system, an opposite phenomenon occurred. The content of quinic acid decreased by 40% when extraction time increased from 30 to 60 min. The reason for the reduction in quinic acid content could be degradation. Namely, due to acoustic cavitation collapse, there is an increase in material cell wall permeability and in the facilitated release of target components. However, prolonged exposure to ultrasound waves and excessive cavitations can cause mechanically or chemically induced degradation of components [22]. Therefore, the extraction efficiency depends on the interaction of physicochemical properties of NADES and the ultrasound process parameters.

The same pattern of dependence of extraction efficiency on time was repeated in the case of catechin, the second most abundant component of the extract (8.84–571.93 µg/g dRSP) (Table 3). The content of catechin scaled up 18-fold when the extraction time was raised from 30 to 90 min and when sugar (LA:Glu:W) was a constituent, whereas it decreased by 25% with the extension of the extraction time when alcohol was a constituent of ternary NADES (LA:Gly:W). However, for the extraction of this flavonoid, the system LA:Gly:W was approximately three times more efficient compared to LA:Glu:W (Figure 2).

Apart from the highest efficiency for quinic acid and catechin, these two systems (LA:Glu:W and LA:Gly:W), due to their polarity which is close to that of water (48.85 ± 1.09 kcal·mol^−1^), were also the most efficient for the extraction of polar acids, such as caffeic acid, *p*-coumaric acid, gallic acid, chlorogenic acid; glycosides, such as quer-3-O-Glc + Gal, lut-7-O-Glc, and kaem-3-O-Glc; and flavonoids, such as naringenin, baicalein, rutin, quercitrin, and quercetin. The most polar system (LA:Glu:W) was the most effective for the recovery of this cyclohexanecarboxylic acid. The less polar BE:LA and LA:Pro were more effective for the recovery of protocatechuic and ferulic acid, and isorhamnetin and amentoflavone, respectively.

The solubility of components is conditioned by their specific structure, the polar part (one or more hydroxyl groups) and the non-polar aromatic ring, and the intermolecular bonds that are established between components and solvents. In general, phenolic components are characterized by a significant number of hydrogen donor and acceptor sites. Therefore, they can establish intermolecular interactions with solvents. Also, the most polar solvents applied had water in their composition. Generally, phenolic acids have good solubility in water due to the presence of hydroxyl groups. This solubility increases with the increasing number of hydroxyl groups. Moreover, most phenolic glycosides are more water-soluble than the corresponding aglycones; therefore, their higher solubility in more polar systems with water is expected. For example, in the structure of gallic acid, there is an aromatic ring with one carboxyl group and three hydroxyl groups; while ferulic acid, as an example, with one hydroxyl group is para-substituted on an aromatic ring which is connected to a highly conjugated side chain. Therefore, gallic acid has a higher affinity for polar solvents (such as water) [23], while ferulic acid was detected only in LA:Pro.

Due to their multicomponent system and improved physicochemical characteristics, NADES have an advantage over pure solvents. It has been shown that DES with water in their composition can dissolve some components much more effectively than pure water. This occurs because the bonds formed between the DES system and the target components are stronger than those with water and can overcome the electrostatic bonds between the material components (prevail over the rest of the solute–solute electrostatic forces) [24].

Also, due to their structure and hydrogen-bonding capacity, polyphenol components are also soluble in less polar solvents such as ethanol and methanol (Bodoira and Maestri, 2020). The polarity of some of the used NADES was close to the polarity of ethanol (51.76 ± 0.54 kcal·mol^−1^) and methanol (51.43 ± 1.13 kcal·mol^−1^).

### 3.2. Principal Component Analysis

**Figure 3:**
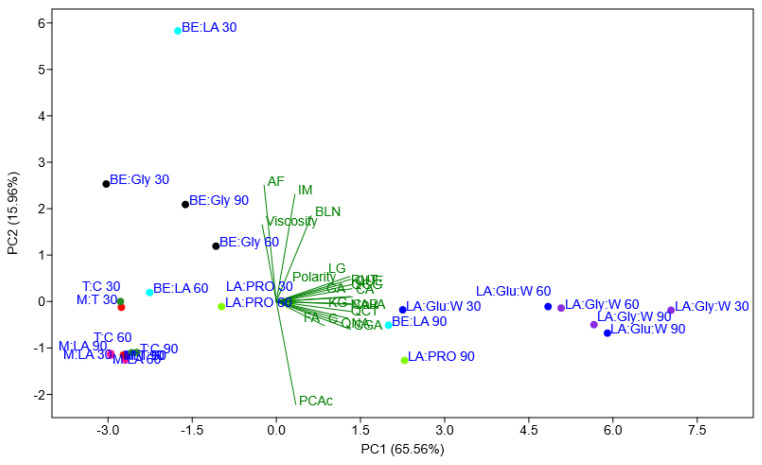
PC1 vs. PC2 correlation bi-plot of analyzed solvent mixtures as scores, and solvent properties (identified compounds, solvent polarity, and viscosity) as loadings. PCAc—Protocatechuic acid; CouA—p-Coumaric acid; GA—Gallic acid; CA—Caffeic acid; QNA—Quinic acid; FA—Ferulic acid; API—Apigenin; BLN—Baicalein; NAR—Naringenin; C—Catechin; QCT—Quercetin; IM—Isorhamnetin; CGA—Chlorogenic acid; RUT—Rutin; KG—Kaempferol 3-O-glucoside; LG—Luteolin 7-O-glucoside; QUE—Quercitrin; QGG—Quer-3-O-Glc + Gal; AF—Amentoflavone.

Figure 3 presents the PCA diagram in PC1 vs. PC2 dimensions, explaining 81.52% of the total variability in the analyzed dataset. The diagram visually depicts the distribution and relationships of data points in the reduced two-dimensional space. This representation offers valuable insights into the underlying patterns and structure within the dataset, facilitating a better understanding of the interaction between the variables and their contributions to the overall variability.

The PCA plot shows a clear and distinct separation of most of the extraction samples based on the applied solvent mixture, regardless of the total extraction time (i.e., 30, 60, or 90 min). This suggests that the choice of solvent combination had a significant impact on the extraction process, influencing the composition of the isolated chemical compounds. The orientation of the loading polarity toward the right in the PCA diagram suggests that the identified features (e.g., identified compounds and solvent polarity) were more pronounced or have higher values in the samples positioned on the right side of the PCA plot. In other words, the right side of the diagram represents samples that exhibited stronger expressions or higher levels of these specific features. Examples include LA:Glu:W and LA:Gly:W systems at all applied extraction times (30, 60, and 90 min).

The viscosity loading is oriented towards the upper-left region in the PCA diagram, indicating that higher values of this feature (viscosity) are associated with samples positioned in that particular region—upper and left. Specifically, the samples represented by BE:LA 30, LA:PRO 30, and BE:Gly at all applied extraction times (30, 60, and 90 min) demonstrated higher viscosity levels, as reflected by their location in the upper-left quadrant of the PCA plot.

Based on the observed trend in the PCA analysis, it can be deduced that solvent mixtures with higher polarity values positioned on the right side exhibited superior extraction performance compared to the solvent blends positioned on the left side of the PCA diagram, which had a lower polarity. Therefore, systems such as T:C, M:T, and M:LA proved to be the least effective. The fact that most of the identified extracted chemical compounds align with the trend of increasing polarity indicates that this property played a crucial role in the extraction process. Solvent mixtures with higher polarity seem to have a greater capacity to extract a wider range of chemical compounds, resulting in improved extraction performance.

Despite LA:Pro and BE:LA resulting in a lower extraction yield at shorter extraction times, the bi-plot clearly demonstrates their improved potential when the extraction duration was extended to 90 min. This finding suggests that these specific solvent mixtures have a time-dependent effect on the extraction process, with longer durations enhancing their extraction efficacy and yielding higher quantities of the targeted compounds.

**Figure 4:**
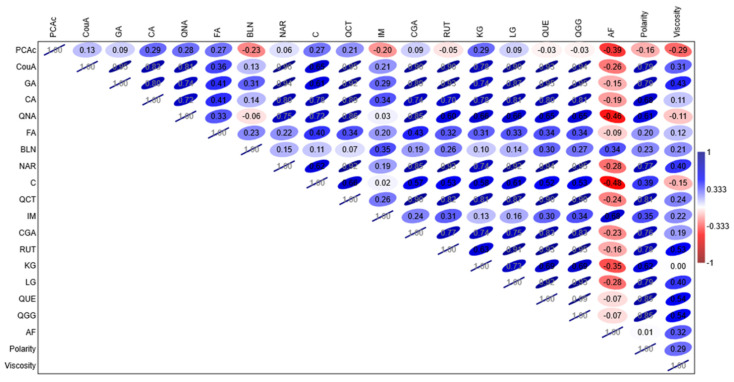
Spearman’s correlation plot; PCAc—Protocatechuic acid; CouA—p-Coumaric acid; GA—Gallic acid; CA—Caffeic acid; QNA—Quinic acid; FA—Ferulic acid; API—Apigenin; BLN—Baicalein; NAR—Naringenin; C—Catechin; QCT—Quercetin; IM—Isorhamnetin; CGA—Chlorogenic acid; RUT—Rutin; KG—Kaempferol 3-O-glucoside; LG—Luteolin 7-O-glucoside; QUE—Quercitrin; QGG—Quer-3-O-Glc + Gal; AF—Amentoflavone.

The obtained Spearman’s correlation plot is shown in Figure 4. Figure 4 indicates that polarity exhibited a high positive correlation (>0.60) with the following chemical compounds: *p*-coumaric acid (CouA), gallic acid (GA), caffeic acid (CA), quinic acid (QNA), naringenin (NAR), quercetin (QCT), chlorogenic acid (CGA), rutin (RUT), kaem-3-O-Glc (KG), lut-7-O-Glc (LG), quercitrin (QUE), and quer-3-O-glc + gal (QGG). However, none of the detected compounds exhibited high positive correlations with solvent viscosity. The highest correlations were observed for QUE and QGG (0.54), RUT (0.53), GA (0.43), NAR, and LG (0.40). Spearman’s correlation plot indicates that there were no significant negative correlations observed, either for polarity or viscosity.

## 4. Conclusions

In this study, it was established that the application of NADES as an extraction medium simultaneously with ultrasonic-assisted extraction can achieve the polyphenol recovery from defatted rosehip seed powder. This approach ensures transformation of defatted seed waste into value-added products—extracts rich in valuable and biologically significant polyphenolic components. The obtained extracts can represent important constituents in the production of various pharmaceutical, cosmetic, and food products. In addition, the process of obtaining extracts implies an environmentally friendly process of extraction with green solvents, without generating solvent waste. Therefore, the established waste utilization procedure fits into the principles of modern sustainable development as they are characterized by waste reduction, increased utilization of natural renewable materials, and the use of safe solvents. The presence of 21 components was found in the obtained extracts; this is the first study in which the seed polyphenolic profile was analyzed in detail. The principal component analysis revealed that the solvent mixture combinations show similar properties in terms of the isolated chemical compounds, with a non-significant effect of the extraction duration on extraction performance. Furthermore, certain correlations between important solvent properties—polarity and viscosity—and the extraction efficiency of specific compounds were observed. Namely, polar and more viscous solvent mixtures were shown to be more successful in extracting the majority of compounds identified in this study. The most efficient systems were ternary lactic acid-based NADES formulations, particularly LA:Glu:W, which ensured obtaining extracts with the highest phenolic composition. Considering that NADES rosehip waste extracts represent a valuable source of polyphenols, the obtained extracts have the potential to be further applied in the sustainable development of different food products.

## Figures and Tables

**Table 1:** NADES composition, abbreviations, and molar ratios.

NADES Composition	Abbreviation	Molar Ratio
Betaine/lactic acid	BE:LA	1:5
Betaine/glycerol	BE:Gly	1:2
Lactic acid/proline	LA:Pro	3:1
Lactic acid/glucose/water	LA:Glu:W	5:1:3
Lactic acid/glycerol/water	LA:Gly:W	3:1:3
Menthol/lauric acid	M:LA	4:1
Thymol/carvacrol	T:C	1:1
Menthol/thymol	M:T	1:1

**Table 2:** Concentration of detected compounds in *R. canina* defatted seed extracts obtained with ultrasound-assisted NADES extraction.

Sample	Quinic Acid	Protocatechuic Acid	*p*-Coumaric Acid	Gallic Acid	Caffeic Acid	Chlorogenic Acid	Ferulic Acid	Kaem-3-O-Glc	Lut-7-O-Glc	Quer-3-O-Glc + Gal
BE:Gly 30	0.44 ± 0.12 ^l^	0.66 ± 0.12 ^h^	<*LoQ*	<*LoQ*	n.d.	n.d.	<*LoQ*	<*LoQ*	<*LoQ*	2.70 ± 1.03 ^f^
BE:Gly 60	125.30 ± 5.14 ^j–l^	3.04 ± 0.40 ^gh^	1.18 ± 0.25 ^a^	4.43 ± 0.55 ^e^	n.d.	n.d.	<*LoQ*	1.61 ± 0.29 ^g^	0.86 ± 0.22 ^a^	9.37 ± 1.77 ^ef^
BE:Gly 90	110.23 ± 7.36 ^j–l^	2.90 ± 0.13 ^gh^	<*LoQ*	4.33 ± 0.81 ^e^	n.d.	n.d.	<*LoQ*	2.53 ± 0.72 ^fg^	0.70 ± 0.14 ^a^	7.07 ± 1.42 ^ef^
BE:LA 30	42.21 ± 2.12 ^kl^	2.09 ± 0.04 ^gh^	<*LoQ*	3.94 ± 0.55 ^e^	<*LoQ*	n.d.	<*LoQ*	1.33 ± 0.99 ^g^	<*LoQ*	5.72 ± 0.55 ^f^
BE:LA 60	22.16 ± 3.21 ^kl^	2.35 ± 0.29 ^gh^	<*LoQ*	<*LoQ*	<*LoQ*	n.d.	<*LoQ*	2.79 ± 1.51 ^fg^	0.58 ± 0.42 ^a^	4.16 ± 1.82 ^f^
BE:LA 90	267.44 ± 7.04 ^ij^	14.19 ± 2.47 ^a–d^	1.52 ± 0.79 ^a^	6.67 ± 1.05 ^c–e^	3.95 ± 0.48 ^a^	n.d.	<*LoQ*	19.40 ± 0.71 ^bc^	1.46 ± 0.15 ^a^	24.44 ± 3.18 ^bc^
LA:Glu:W 30	5650.28 ± 41.15 ^e^	7.76 ± 0.91 ^ef^	2.05 ± 1.05 ^a^	8.12 ± 1.22 ^cd^	<*LoQ*	1.33 ± 0.58 ^a^	<*LoQ*	13.77 ± 1.05 ^cd^	1.43 ± 0.58 ^a^	20.47 ± 2.51 ^cd^
LA:Glu:W 60	13,895.34 ± 152.89 ^b^	10.33 ± 1.13 ^c–e^	2.15 ± 0.71 ^a^	8.29 ± 0.17 ^cd^	4.08 ± 1.21 ^a^	1.32 ± 0.47 ^a^	<*LoQ*	21.43 ± 4.08 ^b^	1.64 ± 0.22 ^a^	28.86 ± 2.41 ^ab^
LA:Glu:W 90	22,428.68 ± 125.63 ^a^	10.03 ± 1.98 ^de^	2.27 ± 0.96 ^a^	9.76 ± 0.73 ^c^	4.14 ± 1.79 ^a^	1.71 ± 0.11 ^a^	<*LoQ*	25.40 ± 1.38 ^ab^	1.70 ± 0.79 ^a^	33.38 ± 1.24 ^a^
LA:Gly:W 30	10,435.87 ± 48.79 ^c^	12.55 ± 3.07 ^b–d^	2.59 ± 0.25 ^a^	17.96 ± 2.08 ^ab^	4.11 ± 0.10 ^a^	2.38 ± 1.03 ^a^	<*LoQ*	28.48 ± 1.87 ^a^	1.51 ± 0.68 ^a^	29.62 ± 1.33 ^ab^
LA:Gly:W 60	6342.17 ± 91.87 ^d^	11.61 ± 1.25 ^b–e^	2.25 ± 0.97 ^a^	16.42 ± 1.52 ^b^	3.76 ± 0.87 ^a^	2.07 ± 0.22 ^a^	<*LoQ*	30.19 ± 3.50 ^a^	1.35 ± 0.72 ^a^	25.37 ± 4.18 ^bc^
LA:Gly:W 90	6331.49 ± 66.87 ^d^	13.88 ± 1.39 ^a–d^	2.22 ± 0.18 ^a^	20.16 ± 1.47 ^a^	4.13 ± 1.32 ^a^	1.63 ± 0.71 ^a^	<*LoQ*	31.24 ± 4.74 ^a^	1.39 ± 0.17 ^a^	26.16 ± 2.49 ^bc^
LA:Pro 30	1068.55 ± 41.30 ^g^	7.41 ± 1.00 ^ef^	1.42 ± 0.49 ^a^	7.01 ± 0.91 ^c–e^	<*LoQ*	0.98 ± 0.18 ^a^	<*LoQ*	4.50 ± 0.55 ^e–g^	1.07 ± 0.44 ^a^	13.61 ± 1.08 ^de^
LA:Pro 60	692.98 ± 87.39 ^h^	5.17 ± 1.04 ^fg^	0.89 ± 0.38 ^a^	5.93 ± 0.35 ^de^	<*LoQ*	1.02 ± 0.51 ^a^	<*LoQ*	4.28 ± 0.88 ^e–g^	<*LoQ*	7.75 ± 2.87 ^ef^
LA:Pro 90	1324.69 ± 18.69 ^f^	18.09 ± 2.17 ^a^	1.41 ± 0.14 ^a^	8.68 ± 0.71 ^cd^	3.53 ± 0.75 ^a^	1.27 ± 0.34 ^a^	1.222 ± 0.19	13.24 ± 2.46 ^cd^	1.25 ± 0.75 ^a^	16.97 ± 3.87 ^d^
M:LA 30	242.55 ± 3.71 ^ij^	14.45 ± 0.88 ^abc^	<*LoQ*	<*LoQ*	<*LoQ*	n.d.	n.d.	<*LoQ*	<*LoQ*	<*LoQ*
M:LA 60	160.76 ± 5.81 ^i–l^	14.91 ± 1.08 ^ab^	<*LoQ*	<*LoQ*	<*LoQ*	n.d.	n.d.	10.10 ± 2.58 ^de^	<*LoQ*	<*LoQ*
M:LA 90	261.13 ± 9.22 ^ij^	14.02 ± 0.55 ^a–d^	<*LoQ*	<*LoQ*	n.d.	n.d.	n.d.	<*LoQ*	<*LoQ*	<*LoQ*
M:T 30	269.20 ± 4.28 ^ij^	<*LoQ*	<*LoQ*	<*LoQ*	n.d.	n.d.	n.d.	8.95 ± 1.89 ^de^	<*LoQ*	<*LoQ*
M:T 60	183.47 ± 14.82 ^i–k^	13.77 ± 2.00 ^b–d^	<*LoQ*	<*LoQ*	n.d.	n.d.	n.d.	9.97 ± 1.34 ^de^	<*LoQ*	<*LoQ*
M:T 90	186.95 ± 3.33 ^i–k^	13.72 ± 1.56 ^b–d^	<*LoQ*	<*LoQ*	n.d.	n.d.	n.d.	8.57 ± 2.29 ^d–f^	<*LoQ*	<*LoQ*
T:C 30	241.53 ± 8.21 ^ij^	<*LoQ*	<*LoQ*	<*LoQ*	n.d.	n.d.	n.d.	<*LoQ*	<*LoQ*	<*LoQ*
T:C 60	303.15 ± 4.98 ^i^	14.02 ± 0.33 ^a–d^	n.d.	<*LoQ*	n.d.	n.d.	n.d.	<*LoQ*	<*LoQ*	<*LoQ*
T:C 90	170.84 ± 4.36 ^i–k^	13.71± 0.75 ^b–d^	n.d.	<*LoQ*	n.d.	n.d.	n.d.	9.63 ± 2.12 ^de^	<*LoQ*	<*LoQ*

Means followed by different letters differ significantly—based on Tukey’s HSD test at *p* < 0.05. <*LoQ*—below quantification limit; n.d.—not detected.

**Table 3:** Concentration of detected phenolics in *R. canina* defatted seed extracts obtained with ultrasound-assisted NADES extraction.

Sample	Apigenin	Baicalein	Naringenin	Catechin	Epicatechin	Epigallocatechin Gallate	Quercetin	Isorhamnetin	Rutin	Quercitrin	Amentoflavone
BE:Gly 30	<*LoQ*	<*LoQ*	n.d.	9.07 ± 1.29 ^k^	n.d.	n.d.	<*LoQ*	1.41 ± 0.56 ^b^	<*LoQ*	2.15 ± 0.97 ^h^	4.09 ± 1.62 ^b^
BE:Gly 60	n.d.	<*LoQ*	0.85 ± 0.26 ^d^	52.34 ± 3.58 ^i^	n.d.	n.d.	<*LoQ*	<*LoQ*	7.21 ± 1.54 ^cd^	10.60 ± 1.84 ^fg^	<*LoQ*
BE:Gly 90	n.d.	<*LoQ*	0.43 ± 0.15 ^d^	39.49 ± 2.98 ^ij^	n.d.	n.d.	<*LoQ*	<*LoQ*	2.77 ± 0.04 ^e–g^	7.74 ± 1.45 ^gh^	<*LoQ*
BE:LA 30	n.d.	<*LoQ*	n.d.	8.84 ± 1.56 k	n.d.	<*LoQ*	<*LoQ*	5.03 ± 1.23 ^a^	2.42 ± 0.47 ^fg^	7.70 ± 0.70 ^gh^	10.69 ± 2.41 ^a^
BE:LA 60	n.d.	<*LoQ*	<*LoQ*	12.49 ± 2.09 k	n.d.	<*LoQ*	<*LoQ*	<*LoQ*	1.73 ± 0.18 ^g^	6.35 ± 1.03 ^gh^	<*LoQ*
BE:LA 90	<*LoQ*	<*LoQ*	1.29 ± 0.39 ^cd^	131.45 ± 5.12 ^fg^	n.d.	<*LoQ*	20.19 ± 1.58 ^bc^	<*LoQ*	4.64 ± 1.02 ^d-g^	36.14 ± 2.48 ^cd^	<*LoQ*
LA:Glu:W 30	<*LoQ*	<*LoQ*	1.80 ± 0.18 ^cd^	10.37 ± 1.09 k	n.d.	<*LoQ*	15.82 ± 0.88 ^cd^	<*LoQ*	5.46 ± 1.00 ^de^	31.92 ± 4.89 ^de^	<*LoQ*
LA:Glu:W 60	<*LoQ*	<*LoQ*	2.86 ± 0.76 ^a–c^	158.81 ± 3.14 ^e^	n.d.	<*LoQ*	23.43 ± 2.14 ^b^	0.68 ± 0.32 ^b^	10.20 ± 0.95 ^ab^	39.15 ± 3.74 ^bc^	<*LoQ*
LA:Glu:W 90	<*LoQ*	<*LoQ*	3.70 ± 1.09 ^a^	192.76 ± 4.18 ^d^	n.d.	<*LoQ*	30.79 ± 1.33 ^a^	<*LoQ*	6.52 ± 0.26 ^cd^	48.18 ± 3.40 ^a^	<*LoQ*
LA:Gly:W 30	<*LoQ*	0.31 ± 0.12 ^a^	1.85 ± 0.98 ^b–d^	571.93 ± 14.78 ^a^	5.433 ^a^	n.d.	22.32 ± 0.45 ^b^	0.84 ± 0.17 ^b^	13.12 ± 2.41 ^a^	45.62 ± 1.55 ^ab^	<*LoQ*
LA:Gly:W 60	<*LoQ*	0.34 ± 0.09 ^a^	2.56 ± 0.63 ^a–c^	468.06 ± 11.08 ^b^	n.d.	n.d.	21.90 ± 0.84 ^b^	<*LoQ*	4.94 ± 1.09 ^def^	39.18 ± 1.87 ^bc^	<*LoQ*
LA:Gly:W 90	<*LoQ*	<*LoQ*	3.46 ± 0.47 ^ab^	434.58 ± 13.55 ^c^	n.d.	<*LoQ*	34.70 ± 1.04 ^a^	1.33 ± 0.78 ^b^	8.80 ± 0.41 ^bc^	34.66 ± 0.74 ^c–e^	<*LoQ*
LA:Pro 30	<*LoQ*	<*LoQ*	0.81 ± 0.14 ^d^	72.62 ± 2.58 ^h^	n.d.	<*LoQ*	12.95 ± 0.22 ^d^	<*LoQ*	6.18 ± 0.61 ^cd^	17.45 ± 2.43 ^f^	<*LoQ*
LA:Pro 60	<*LoQ*	<*LoQ*	0.56 ± 0.29 ^d^	34.19 ± 0.78 ^j^	n.d.	<*LoQ*	11.56 ± 2.70 ^d^	<*LoQ*	2.56 ± 0.22 ^e–g^	11.69 ± 2.54 ^fg^	<*LoQ*
LA:Pro 90	n.d.	<*LoQ*	0.48 ± 0.17 ^d^	151.62 ± 2.19 ^e^	n.d.	n.d.	19.40 ± 3.14 ^bc^	<*LoQ*	4.42 ± 0.74 ^de^	28.44 ± 1.47 ^e^	<*LoQ*
M:LA 30	<*LoQ*	n.d.	<*LoQ*	39.18 ± 1.14 ^ij^	n.d.	n.d.	<*LoQ*	<*LoQ*	<*LoQ*	<*LoQ*	<*LoQ*
M:LA 60	<*LoQ*	n.d.	<*LoQ*	39.74 ± 0.75 ^ij^	n.d.	n.d.	<*LoQ*	<*LoQ*	<*LoQ*	<*LoQ*	<*LoQ*
M:LA 90	<*LoQ*	n.d.	<*LoQ*	39.32 ± 0.87 ^ij^	n.d.	n.d.	<*LoQ*	n.d.	<*LoQ*	<*LoQ*	<*LoQ*
M:T 30	<*LoQ*	n.d.	<*LoQ*	121.23 ± 3.17 ^fg^	n.d.	n.d.	<*LoQ*	<*LoQ*	<*LoQ*	<*LoQ*	<*LoQ*
M:T 60	<*LoQ*	n.d.	<*LoQ*	39.47 ± 0.75 ^ij^	n.d.	n.d.	<*LoQ*	n.d.	<*LoQ*	<*LoQ*	<*LoQ*
M:T 90	<*LoQ*	n.d.	<*LoQ*	38.79 ± 1.13 ^ij^	n.d.	n.d.	<*LoQ*	n.d.	<*LoQ*	<*LoQ*	<*LoQ*
T:C 30	<*LoQ*	n.d.	<*LoQ*	115.97 ± 4.41 ^g^	n.d.	n.d.	<*LoQ*	n.d.	<*LoQ*	<*LoQ*	<*LoQ*
T:C 60	<*LoQ*	n.d.	<*LoQ*	134.46 ± 2.87 ^f^	n.d.	n.d.	<*LoQ*	n.d.	<*LoQ*	<*LoQ*	<*LoQ*
T:C 90	<*LoQ*	n.d.	<*LoQ*	38.86 ± 1.16 ^ij^	n.d.	n.d.	<*LoQ*	n.d.	<*LoQ*	<*LoQ*	<*LoQ*

Means followed by different letters differ significantly—based on Tukey’s HSD test at *p* < 0.05. <*LoQ*—below quantification limit; n.d.—not detected.

## Data Availability

The data used to support the findings of this study can be made available by the corresponding author upon request.

## Supplementary Materials

The following supporting information can be downloaded at: https://www.mdpi.com/article/10.3390/foods12193655/s1, Table S1: NADES composition, abbreviation, molar ratio, polarity, and viscosity at 60°C; Table S2. Validation results—linear fit parameters (r^2^—the coefficient of determination, and limit of linearity), limit of detection (LOD) and limit of quantification (LOQ).
