# Supplementary material for: Recovery of Polyphenols from Rosehip Seed Waste Using Natural Deep Eutectic Solvents and Ultrasonic Waves Simultaneously
Source: Foods. 2023 Oct 3;12(19):3655. doi: 10.3390/foods12193655 (PMC10572640; doi:10.3390/foods12193655)
Supplement: Supplementary file 1 [file foods-12-03655-s001.zip › foods-2644963-supplementary.pdf]

Supplementary Materials

Recovery of Polyphenols from Rosehip Seed Waste Using Natural Deep Eutectic Solvents and Ultrasonic Waves Simultaneously

Aleksandra Gavarić [1], Kristian Pastor [1], Nataša Nastić [1], Senka Vidović [1], Nemanja Živanović [2], Nataša Simin [2], Ana Rita C. Duarte [3] and Jelena Vladić [3,*]

[1] Faculty of Technology, University of Novi Sad, Bulevar cara Lazara 1, 21000 Novi Sad, Serbia; cvejina@uns.ac.rs (A.G.); kristian.pastor@uns.ac.rs (K.P.); natasa.nastic@uns.ac.rs (N.N.)
[2] Faculty of Sciences, University of Novi Sad, Trg Dositeja Obradovića 3, 21000 Novi Sad, Serbia; nemanja.zivanovic@dh.uns.ac.rs (N.Ž.)
[3] LAQV, REQUIMTE, Chemistry Department, NOVA School of Science and Technology, 2829-516 Caparica, Portugal
* Correspondence: jelenavladic@fct.unl.pt

Table S1. NADES composition, abbreviation, molar ratio, polarity, and viscosity at 60°C

NADES composition	Abbreviation	Molar ratio	E_{NR} (kcal·mol^{-1})	η (mPa·s)
Betaine:lactic acid	BE:LA	1:5	49.18±2.06	34.80±2.51
Betaine:glycerol	BE:Gly	1:2	49.98±0.79	227.33±4.66
Lactic acid:proline	LA:Pro	3:1	49.52±1.15	76.17±1.72
Lactic acid:glucose:water	LA:Glu:W	5:1:3	47.86±2.31	19.77±0.59
Lactic acid:glycerol:water	LA:Gly:W	3:1:3	48.00±0.58	6.77±0.06
Menthol:lauric acid	M:LA	4:1	53.18±0.58	6.45±0.07
Thymol:carvacrol	T:C	1:1	49.58±1.06	4.07±0.47
Menthol:thymol	M:T	1:1	50.99±1.12	5.95±0.07

Table S2. Validation results – linear fit parameters (r^2 - the coefficient of determination, and limit of linearity), limit of detection (LOD) and limit of quantification (LOQ)

**Compound**	r^2	**Limit[a] (μg/mL)**	**LOD (μg/mL)**	**LOQ (μg/mL)**
Quinic acid	0.995	25[b]	0.01	0.01
Protocatechuic acid	0.996	3.1	0.004	0.004
p-Coumaric acid	0.995	3.1	0.004	0.004
Gallic acid	0.990	1.6	0.02	0.02
Caffeic acid	0.989	3.1	0.006	0.006
Chlorogenic acid	0.994	6.2	0.007	0.007
Ferulic acid	0.992	6.2	0.01	0.01
Kaemferol-3-*O*-Glc	0.997	3.1	0.004	0.004
Luteolin-7-*O*-Glc	0.997	3.1	0.005	0.005
Quercetin-3-*O*-Glc+Gal	0.995	3.1	0.006	0.006
Apigenin	n/a	n/a	0.01	0.01
Baicalein	0.993	6.2	0.03	0.03
Naringenin	0.993	3.1	0.007	0.007
Catechin	0.996	6.2	0.05	0.05
Epicatechin	0.992	6.2	0.06	0.06
Epigallocatechin gallate	0.996	1.2	>0.1[c]	>0.1[c]
Quercetin	0.980	1.6	>0.1[c]	>0.1[c]
Isorhamnetin	0.990	1.6	0.02	0.02
Rutin	0.996	6.2	0.003	0.003
Quercitrin	0.994	1.2	0.003	0.003
Amentoflavone	n/a	n/a	0.005	0.005

n/a – not aplicable since response was nonlinear in the entire evaluated range.

[a] Linearity limit - the highest calibration level used for linear regression.

[b] The highest concentration tested; the actual limit may be higher.

[c] Reliable quantification limit was above the highest concentration examined but was not further evaluated.
